# CMR quantitation of change in mitral regurgitation following transcatheter aortic valve replacement (TAVR): impact on left ventricular reverse remodeling and outcome

**DOI:** 10.1007/s10554-018-1441-y

**Published:** 2018-09-04

**Authors:** Pei G. Chew, Laura E. Dobson, Pankaj Garg, Timothy A. Fairbairn, Tarique A. Musa, Akhlaque Uddin, Peter P. Swoboda, James R. Foley, Graham J. Fent, Louise A. E. Brown, Sebastian Onciul, Sven Plein, Daniel J. Blackman, John P. Greenwood

**Affiliations:** 1 Multidisciplinary Cardiovascular Research Centre (MCRC) & Leeds Institute of Cardiovascular and Metabolic Medicine (LICAMM), University of Leeds, Leeds, LS2 9JT UK; 2 Leeds Teaching Hospitals NHS Trust, Leeds, UK

**Keywords:** Mitral regurgitation, Mitral insufficiency, Transcatheter aortic valve replacement, Cardiovascular magnetic resonance, Late gadolinium enhancement

## Abstract

Current echocardiographic data reporting the impact of concomitant mitral regurgitation (MR) on outcome in patients who undergo transcatheter aortic valve replacement (TAVR) are conflicting. Using cardiovascular magnetic resonance (CMR) imaging, this study aimed to assess the impact of MR severity on cardiac reverse remodeling and patient outcome. 85 patients undergoing TAVR with CMR pre- and 6 m post-TAVR were evaluated. The CMR protocol included cines for left (LV) and right ventricular (RV) volumes, flow assessment, and myocardial scar assessment by late gadolinium enhancement (LGE). Patients were dichotomised according to CMR severity of MR fraction at baseline (‘non-significant’ vs ‘significant’) and followed up for a median duration of 3 years. Forty-two (49%) patients had ‘significant MR’ at baseline; they had similar LV and RV size and function compared to the ‘non-significant MR’ group but had greater LV mass at baseline. In those with significant MR at baseline, 77% (n = 32) had a reduction in MR post-TAVR, moving them into the ‘non-significant’ category at 6-months, with an overall reduction in MR fraction from 34 to 17% (p < 0.001). Improvement in MR was not associated with more favourable cardiac reverse remodeling when compared with the ‘non-improvers’. Significant MR at baseline was not associated with increased mortality at follow-up. Significant MR is common in patients undergoing TAVR and improves in the majority post-procedure. Improvement in MR was not associated with more favourable LV reverse remodeling and baseline MR severity was not associated with mortality.

**Electronic supplementary material:**

The online version of this article (10.1007/s10554-018-1441-y) contains supplementary material, which is available to authorized users.

## Introduction

Transcatheter aortic valve replacement (TAVR) has been shown to reduce mortality and improve patient symptoms and quality of life [1–3], and is an alternative to surgery in intermediate and high-risk patients with severe aortic stenosis (AS) [4]. Whilst moderate or severe mitral regurgitation (MR) is seen in up to 48% of patients undergoing TAVR, it is often left untreated [5–7]. Current literature reporting the impact of concomitant MR on outcome in patients who undergo TAVR are conflicting and are mainly based on echocardiographic data; which can be limited by poor acoustic windows, eccentric jets and geometric assumptions [8]. Cardiovascular magnetic resonance (CMR) imaging is able to quantify MR with high accuracy and reproducibility using a combination of left ventricular (LV) volumetric measurements and aortic flow quantification [9–11]. Tissue characterization is a further unique strength of CMR, offering non-invasive detection of myocardial fibrosis [12]. In a TAVR population however, quantitative serial assessment of MR by CMR has never been specifically studied, despite its objectiveness, reproducibility and accuracy.

The aims of this study were to (1) to quantitate the change in MR severity at 6-months post-TAVR using CMR, (2) identify determinants of improvement in MR and its association with LV reverse remodeling, (3) assess the clinical impact of MR on the outcomes of patients undergoing TAVR.

## Methods

### Study design and population

In this prospective study, 109 patients with severe AS undergoing TAVR between April 2009 and September 2015 at a single tertiary centre (Leeds General Infirmary, Leeds, UK) were evaluated. Severe AS was defined as an echocardiographically derived aortic valve area of ≤ 1.0 cm^2^, peak aortic velocity of > 4 m/s or mean pressure gradient of > 40 mmHg. Decision for TAVR intervention was taken by a multidisciplinary heart team in accordance with international guidance. Exclusion criteria included any contraindications to CMR. Baseline clinical, demographic and echocardiographic data were recorded for all patients. CMR scans were performed at baseline and 6-months post-TAVR.

All patients were followed up for a median duration of 3 years and their long-term outcomes were evaluated. Mortality data were obtained from the Office of National Statistics, UK. All patients provided written informed consent. The study was approved by the National Research Ethics Service (08/H1307/106) and complied with the Declaration of Helsinki.

### TAVR

Patients underwent a standard work-up for TAVR which included transoesophageal echocardiography and invasive coronary angiography, with the addition of cardiac computed tomography after 2014. Coronary revascularisation was only performed in those with critical proximal lesions or symptomatic angina. TAVR was performed under general or local anaesthetic using the self-expanding Medtronic CoreValve (Medtronic Inc, Minneapolis, MIN) or the mechanically expanded Boston Lotus valve (Boston Scientific Corporation, Natick, MA) via the femoral or subclavian route by two experienced, high-volume operators. All patients received heparin to maintain an activated clotting time > 250 s and were treated with dual antiplatelet therapy (aspirin and clopidogrel) for 3–6 months after the procedure.

### CMR protocol

Details of the CMR pulse sequence have been previously described [13]. Briefly, identical baseline preoperative and 6-month postoperative scans were performed on a 1.5T MRI system (Intera, Philips Healthcare, Best, Netherlands or Avanto, Siemens Medical Systems, Erlangen, Germany); same scanner vendor used at baseline and 6-months. Multi-slice, multi-phase cine imaging was performed using a standard steady-state free precession pulse sequence in the short axis (repetition time (TR) 3 ms, echo time (TE) 1.7 ms, flip angle 60°, SENSE factor 2, 8 mm thickness, 0 mm gap, 30 phases, matrix 192 × 192, typical field of view (FOV) 340 mm) to cover the entire left and right ventricle. Through-plane velocity encoded (VENC) phase contrast imaging was performed at the aortic sinotubular junction (VENC 250–500 cm/s, retrospective gating, slice thickness 6 mm, 40 phases, FOV 340 mm) or just above the valve prosthesis post-replacement. VENC was typically set at 400–500 cm/s on the baseline scan and 250 cm/s post-procedure. If aliasing occurred at the pre-set VENC, sequential phase contrast imaging was performed at increasing VENC settings until the aliasing artefact had disappeared.

Late gadolinium enhancement (LGE) imaging (10–12 short axis slices, 10 mm thickness, matrix 240 × 240, typical FOV 340 mm) was performed following a Look-Locker sequence (inversion time scout), 10 min after the administration of 0.2 mmol/kg of gadoteric acid (Dotarem, Guerbet, Villepinte). Four chamber, two chamber and left ventricular outflow tract (LVOT) views were also obtained as standard. Cross cuts and phase swap imaging were used where necessary for further clarification of the presence/absence of LGE.

### CMR analysis

CMR analysis was performed by two experienced CMR operators (LED, PGC) blinded to clinical details, using dedicated computer software (CVI^42^, Circle Cardiovascular Imaging, Calgary, Alberta, Canada). LV endocardial and epicardial borders were manually contoured (with trabeculation and papillary muscles excluded) at end-diastole and end-systole to allow the calculation of ventricular volumes (summation of discs methodology) and LV mass [epicardial volume − endocardial volume multiplied by myocardial density (1.05 g/cm^3^)]; values were indexed to body surface area. Left atrial volume was calculated using the formula:

$$\frac{{8\,(A{2_{Ch}})(A{4_{Ch}})}}{{3\uppi {\text{L}}}}$$

where A2_Ch_ and A4_Ch_ refer to the left atrial area in the two-chamber and four-chamber views respectively, and L is the shorter of the two left atrial length measurements. Aortic flow was quantified using cross-sectional phase contrast images with contouring of the aortic lumen to provide aortic forward flow data. MR fraction (%) was quantified using the equation:

$$\frac{{{\text{LV}}~{\text{stroke}}~{\text{volume}}-{\text{aortic}}~{\text{forward}}~{\text{flow}}~{\text{volume}}}}{{{\text{LV}}~{\text{stroke}}~{\text{volume}}}}~ \times ~100$$

‘Significant MR’ was defined as MR fraction > 25% and ‘non-significant MR’ was defined as MR fraction ≤ 25% [10]. For the purpose of this study, ‘significant MR’ represented moderate-severe/severe categories and ‘non-significant MR’ comprised categories of trivial/mild/moderate as per CMR classification. Changes in the MR severity were assessed between the baseline and 6-month post-procedure scans. Those with a reduction in MR severity grade from ‘significant’ to ‘non-significant’ category were classified as ‘improvers’, and those without (i.e. MR worsened or unchanged) were labelled as ‘non-improvers’.

LGE images were reviewed for the presence or absence of hyper-enhancement, which was then classified as either non-infarct pattern (myocardial fibrosis), infarct pattern, or mixed pattern. The number and location of segments containing LGE were classified according to the American Heart Association (AHA) 17-segment model. Myocardial fibrosis was defined as a region of LGE with signal enhancement > 5 SD of the signal intensity of non-enhanced myocardium.

### Statistical analysis

All statistical analysis was performed using the SPSS V.21.0 (IBM Corp., New York, USA). Continuous variables are expressed as mean ± SD or median (interquartile range, IQR) in cases of skewed distributions. Categorical variables are expressed as frequencies and percentages. Data were tested for normality using the Shapiro–Wilks test. For normally distributed continuous data, two-tailed unpaired Student’s *t* tests were used for comparisons between groups, and paired Students *t* tests were used for intra-group comparisons. For non-normally distributed data, Mann–Whitney *U*-test was used. The Chi-Squared test was used for comparing categorical variables.

In order to assess the correlation between dependent and independent variables, Pearson’s correlation coefficients were used. Two-sided P values < 0.05 were considered statistically significant. Univariate analysis was used to determine predictive factors for MR improvement. Variables with a univariate p < 0.1 were entered into multi-variable regression analysis. Cumulative survival was analyzed with Kaplan–Meier methodology and log-rank test.

## Results

### Patients and baseline characteristics

From 109 patients with a baseline scan, those with a permanent pacemaker (n = 8), severe aortic regurgitation (n = 5) or who had an incomplete scan (n = 1), were excluded from analysis. Eight patients declined follow up and 2 patients died prior to their 6 month scan. 85 patients with paired CMR scans (55% male gender, mean age 80 ± 7 years) who underwent TAVR for severe AS were included in the final data analysis. Basic demographics and clinical data can be seen in Table 1.

**Table 1:** Baseline demographics in all patients, ‘non-significant’ and ‘significant’ MR groups

	All patients	Non-significant MR (n = 43)	Significant MR (n = 42)	p value
Age at TAVR	80.2 ± 4.9	80.1 ± 7.2	80.2 ± 7.5	0.93
Male sex, n (%)	47 (55)	23 (53)	24 (57)	0.73
Logistic euroscore	19.8 ± 13.1	19.6 ± 13.1	20.0 ± 13.2	0.80
Euroscore II	5.45 ± 4.42	5.4 ± 4.4	5.5 ± 4.4	0.80
STS mortality	4.8 ± 2.97	5.1 ± 3.3	4.4 ± 2.5	0.20
STS morbidity	23.2 ± 8.42	23.7 ± 8.3	22.7 ± 8.5	0.50
HTN	44.7%	46.5%	42.8%	0.70
DM	20.0%	20.9%	19.0%	0.80
AF	21.2%	25.5%	16.6%	0.30
MI	22.4%	20.9%	23.8%	0.80
CABG	29.4%	20.9%	38.0%	0.08
PCI	25.9%	25.5%	26.1%	0.90
PVD	21.2%	23.2%	19.0%	0.60
CVA	15.3%	13.9%	16.6%	0.70
PHT	37.6%	30.2%	45.2%	0.15
Revascularization pre-TAVR	8 (9)	4 (9)	4 (10)	0.63
Aortic valve parameters (echocardiogram)				
AVAi	0.33 ± 0.84	0.33 ± 0.09	0.33 ± 0.07	0.99
AV max velocity	4.7 ± 0.51	4.6 ± 0.51	4.8 ± 0.47	0.02
AV mean PG	49.7 ± 11.6	47.5 ± 10.8	51.9 ± 12.1	0.07

Data as mean ± SD, n (%)

*AF* atrial fibrillation, *AV* aortic valve, *AVAi* aortic valve area (indexed), *CABG* coronary artery bypass graft, *CVA* cerebrovascular attack, *DM* diabetes mellitus, *HTN* hypertension, *MI* myocardial infarction, *PCI* percutaneous coronary intervention, *PG* pressure gradient, *PHT* pulmonary hypertension, *PVD* peripheral vascular disease, *STS* society of thoracic surgery, *TAVR* transcatheter aortic valve replacement

In total, 42/85 (49%) patients were classified as having ‘significant MR’, and 43/85 (51%) as ‘non-significant MR’. Those with ‘significant’ MR had a mitral regurgitant volume of 34.5 ± 9.9 ml and a regurgitant fraction of 34.2 ± 5.5%. Comparatively, those with ‘significant’ MR had a greater echocardiographically measured aortic peak forward flow velocity (4.8 ± 0.47 m/s vs 4.6 ± 0.51 m/s, p = 0.02), although mean pressure gradient and aortic valve area did not differ significantly. The ‘significant MR’ group (n = 42) had similar LV and right ventricular (RV) cavity size and function but had greater LV mass at baseline compared to the ‘non-significant MR’ group (Table 2). Those with significant MR also had more aortic regurgitation (aortic regurgitant fraction 13.3 ± 6.3% vs 9.5 ± 8.4%, p = 0.008) by CMR. The presence of LGE was not statistically different between groups [‘significant’ 21.4% (n = 9) vs ‘non-significant’ 34.8% (n = 15), p = 0.188].

**Table 2:** Baseline CMR characteristics of patients in all patients, ‘non-significant’ and ‘significant’ MR groups

	All patients	Non-significant MR (n = 43)	Significant MR (n = 42)	p value
LV mass (g)	138.2 ± 35.3	127.5 ± 31	149 ± 32.9	0.007
LV mass index (g/m^2^)	76.1 ± 18.3	73.5 ± 16.5	83.3 ± 23.3	0.01
LVEDV (ml)	179 ± 49.3	170 ± 44.2	183 ± 45.3	0.33
LVESV (ml)	84.2 ± 43.5	86.7 ± 50.8	81.7 ± 34.9	0.59
LVEF (%)	54.8 ± 12.2	52.5 ± 13.3	56.3 ± 11	0.14
RVEDV (ml)	139.9 ± 36.0	135.6 ± 32.1	144.3 ± 39.5	0.27
RVEF (%)	54.2 ± 9.5	53.5 ± 10.7	55.0 ± 8.8	0.46
LA volume (ml)	131.8 ± 45.0	130.9 ± 51.4	132.8 ± 38.1	0.85
LA volume index (ml/m^2^)	72.8 ± 24.9	73.0 ± 28.8	72.6 ± 20.7	0.94
MR volume (ml)	22.4 ± 15.0	10.3 ± 8.1	34.5 ± 9.9	< 0.001
MR fraction (%)	22.6 ± 13.3	11.4 ± 9.0	34.2 ± 5.5	< 0.001
Classifications of LGE, n (%)				
None	24 (28)	15 (35)	9 (21)	
Infarct pattern	19 (22)	10 (23)	9 (21)	
Non-infarct pattern	33 (39)	14 (33)	19 (45)	
Mixed	4 (5)	2 (5)	2 (5)	
Not done	5 (6)	2 (5)	3 (7)	
Presence of LGE n, (%)				
LGE present	56 (66)	15 (35)	9 (21)	0.188
LGE absent	24 (28)	26 (60)	30 (71)	
LGE not done	5 (6)	2 (5)	3 (7)	

Data as mean ± SD, n (%)

*LA* left atrial, *LGE* late gadolinium enhancement, *LVEDV* left ventricular end-diastolic volume, *LVEF* left ventricular ejection fraction, *LVESV* left ventricular end-systolic volume, *LVSV* left ventricular stroke volume, *MR* mitral regurgitation, *RVEDV* right ventricular end diastolic volume, *RVEF* right ventricular ejection fraction

### Cardiac reverse remodeling following TAVR

Following TAVR, all patients sustained a significant decrease in their peak aortic valve gradient from 41 ± 16 mmHg to 18 ± 10 mmHg (p < 0.001) by CMR. At 6 months, compared to baseline, there were significant reductions in LV end-diastolic volumes (p < 0.001), LV end-systolic volumes (p = 0.006), and LV mass (p < 0.001) (Table 3). Global LV and RV ejection fractions however did not change. In addition, left atrial volumes significantly reduced post-TAVR intervention (Table 3).

**Table 3:** CMR parameters pre- and post-TAVR interventions in all patients

All patients	Baseline n = 85	6 m follow up n = 85	p value
LV mass (g)	138.2 ± 35.3	109.9 ± 31	< 0.001
LVEDV (ml)	179 ± 49.3	166.4 ± 44.2	< 0.001
LVESV (ml)	84.2 ± 43.5	75.7 ± 35.6	0.006
LVSV (ml)	94.5 ± 22.5	90.7 ± 18.7	0.04
LVEF (%)	54.8 ± 12.2	56.3 ± 10.6	0.10
RVSV (ml)	74.3 ± 18.4	78.7 ± 20.4	0.04
RVEF (%)	54.2 ± 9.5	55.4 ± 10.1	0.20
LA volume (ml)	131.8 ± 45.0	119.1 ± 41.3	< 0.001
MR volume (ml)	22.4 ± 15.0	13.7 ± 12.9	< 0.001
MR fraction (%)	22.6 ± 13.3	14.5 ± 12.4	< 0.001
MR classifications (n)			
Mitral regurgitation %			
MR none (0%)	8	14	
MR mild (5–15%)	20	35	
MR moderate (16–25%)	19	20	
MR moderate-severe (26–48%)	38	16	< 0.001
Classifications of LGE, n (%)			
None	24 (28)	28 (33)	
Infarct pattern	19 (22)	23 (27)	
Non-infarct pattern	33 (39)	24 (28)	0.23
Mixed	4 (5)	3 (4)	
Not done	5 (6)	7 (8)	
Presence of LGE, n (%)			
LGE present	56 (66)	50 (59)	
LGE absent	24 (28)	28 (33)	
LGE not done	5 (6)	7 (8)	

Data as mean ± SD, n (%)

*LA* left atrial, *LGE* late gadolinium enhancement, *LVEDV* left ventricular end-diastolic volume, *LVEF* left ventricular ejection fraction, *LVESV* left ventricular end-systolic volume, *LVSV* left ventricular stroke volume, *MR* mitral regurgitation, *RVEF* right ventricular ejection fraction, *RVSV* right ventricular stroke volume

**Fig. 1:**
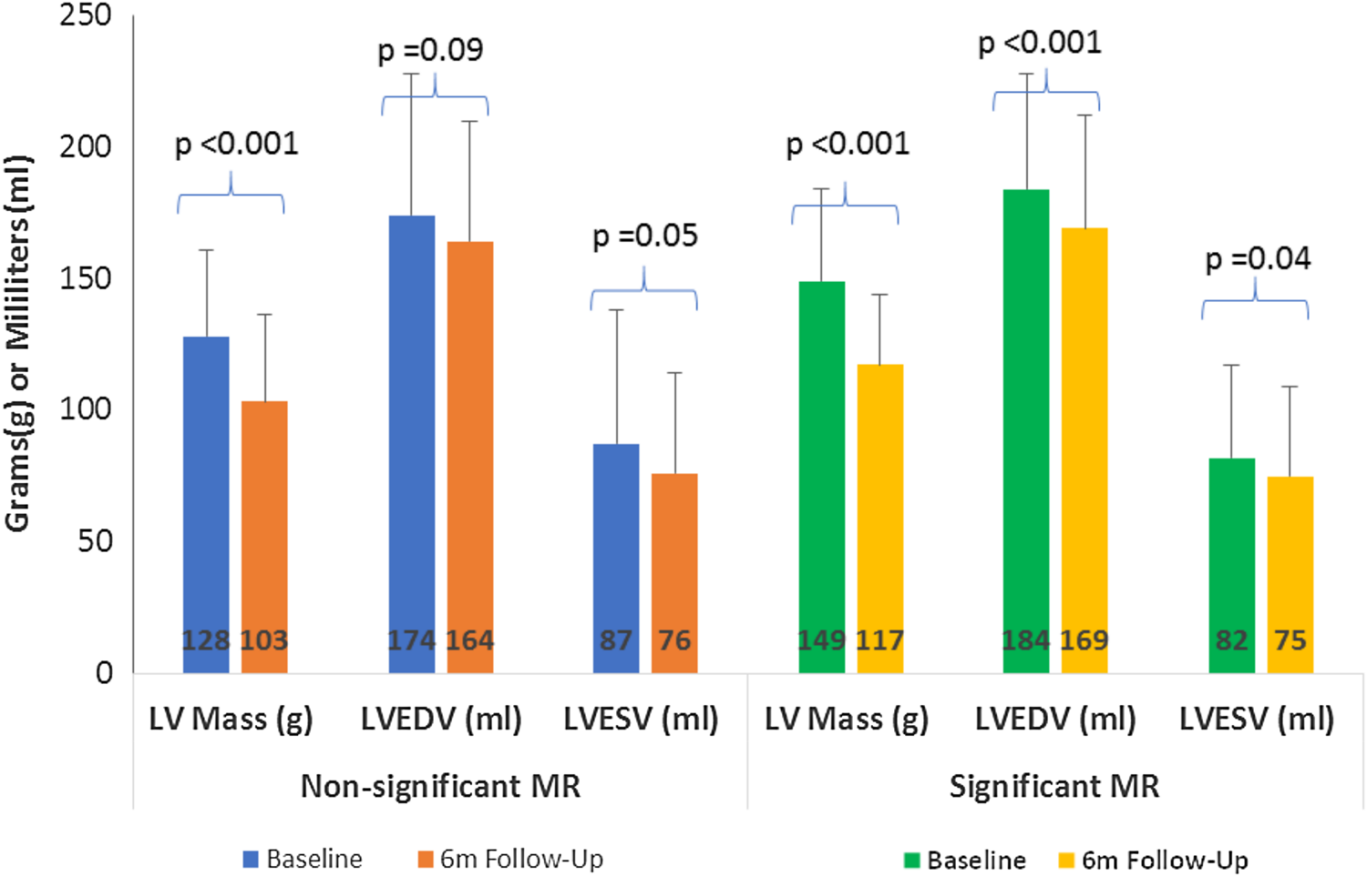
CMR characteristics at baseline and 6-months for ‘significant’ and ‘non-significant’ MR groups. *LVEDV* left ventricular end-diastolic volume, *LVESV* left ventricular end-systolic volume, *MR* mitral regurgitation

The ‘significant’ MR group had a greater degree of reduction in both MR regurgitant volumes (− 19 ± 14 ml vs 1 ± 13 ml, p < 0.001) and MR fraction (− 17 ± 13% vs 1 ± 14%, p < 0.001). No significant change in LV ejection fraction (0.2 ± 8% vs 3 ± 9%, p = 0.15), RV ejection fraction (2 ± 9% vs 1 ± 9%, p = 0.54) or LV mass (− 32 ± 19 g vs − 25 ± 18 g, p = 0.07) were seen between groups. Those with significant MR experienced a greater reduction in LV end-diastolic (p < 0.001) and end-systolic volumes (p = 0.04) when compared to the ‘non-significant’ MR group (Fig. 1).

### Changes in MR fraction in the ‘significant MR’ group

**Fig. 2:**
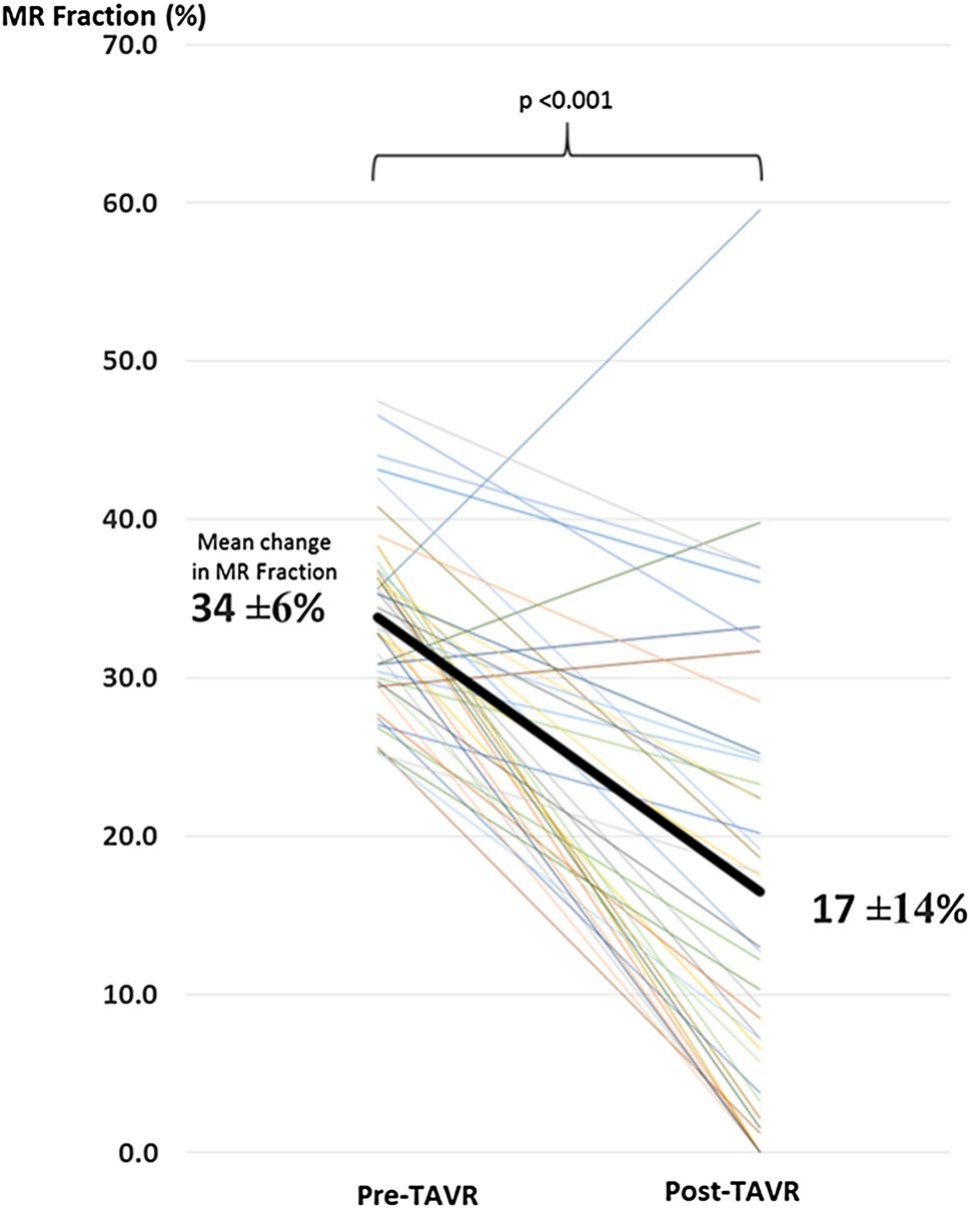
Change in MR fraction (%) in the ‘significant MR’ group post-TAVR. *MR* mitral regurgitant, *TAVR* transcatheter aortic valve replacement

In those with significant MR at baseline (n = 42), 77% (n = 32) had a significant reduction in MR, moving them into the ‘non-significant’ category at 6 months, with an overall reduction in MR fraction from 34 ± 6% to 17 ± 14% (p < 0.001) (Fig. 2).

### Changes in haemodynamics and cardiac reverse remodeling according to MR ‘improver’ and ‘non-improver’ status

**Fig. 3:**
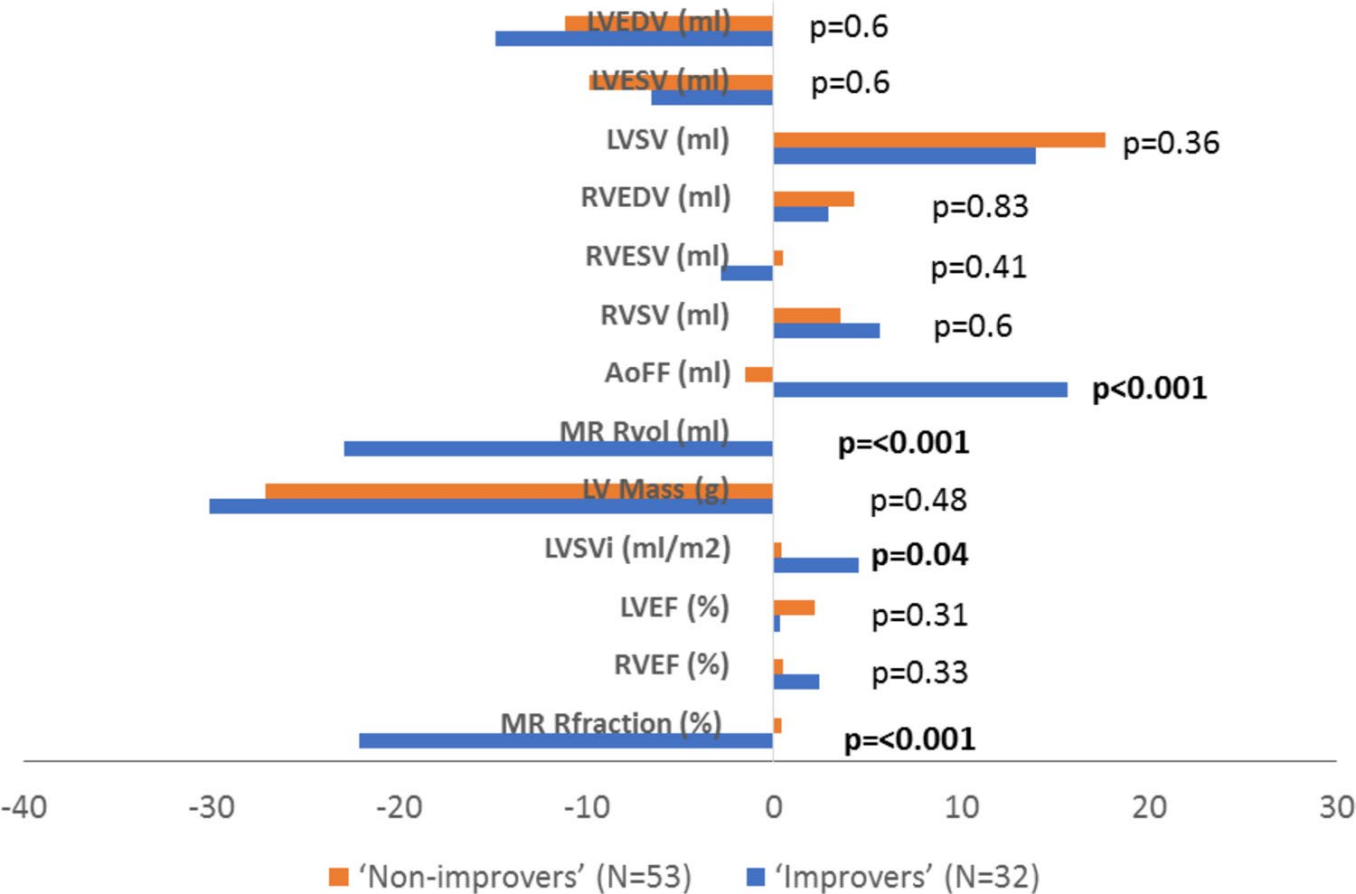
Change in cardiac reverse remodeling parameters in ‘improvers’ and ‘non-improvers’. *AoFF* aortic forward flow, *LVEDV* left ventricular end-diastolic volume, *LVEF* left ventricular ejection fraction, *LVESV* left ventricular end-systolic volume, *LVSV* left ventricular stroke volume, *LVSVi* left ventricular stroke volume indexed, *MR* mitral regurgitation, *Rfraction* regurgitant fraction, *RVESV* right ventricular end-systolic volume, *RVEDV* right ventricular end-diastolic volume, *RVEF* right ventricular ejection fraction, *Rvol* regurgitant volume, *RVSV* right ventricular stroke volume

From the total study population, MR significantly improved in 38% (n = 32) of patients 6-months post-TAVR, and worsened/unchanged in 62% (n = 53) of patients. At follow up, the ‘improvers’ group, but not the ‘non-improvers’, had a significant improvement in LV stroke volume index (p = 0.04) and a greater increase in aortic forward flow (p < 0.001). Improvement in MR however was not associated with more favourable cardiac LV reverse remodeling compared with the ‘non-improvers’ (Fig. 3).

In the ‘improvers’ group, 72% (n = 23) had presence of LGE, 22% (n = 7) had no LGE and LGE imaging was not performed in 6% (n = 2) due to severe renal impairment. In those with LGE, the pattern of LGE was non-infarct pattern in 61% (n = 14), infarct pattern in 35% (n = 8), and mixed in 4% (n = 1). The presence of LGE at baseline was associated with a greater reduction in MR fraction at 6-months following TAVR intervention (− 11 ± 16% vs 0.2 ± 16%, p = 0.01).

### Other factors associated with MR improvement

Univariate regression analysis was conducted to look for any clinical or CMR factors associated with MR reduction following TAVR. The following variables were tested: baseline demographics, baseline and 6 months- LV and RV ejection fraction, mass, and volumes; pre-treatment and post-treatment mean trans-aortic gradient (see Online Supplementary Appendix). A higher baseline RV ejection fraction or RV stroke volume, and a greater reduction in LV end-diastolic pressure (LVEDP) post-TAVR were all significantly associated with MR improvement. A lower aortic forward flow at baseline was also associated with the reduction in MR. Multivariate predictors of improved MR following TAVR intervention were pre-operative absence of atrial fibrillation, a higher RV stroke volume and a lower aortic forward flow at baseline.

### Impact of MR on mortality

**Fig. 4:**
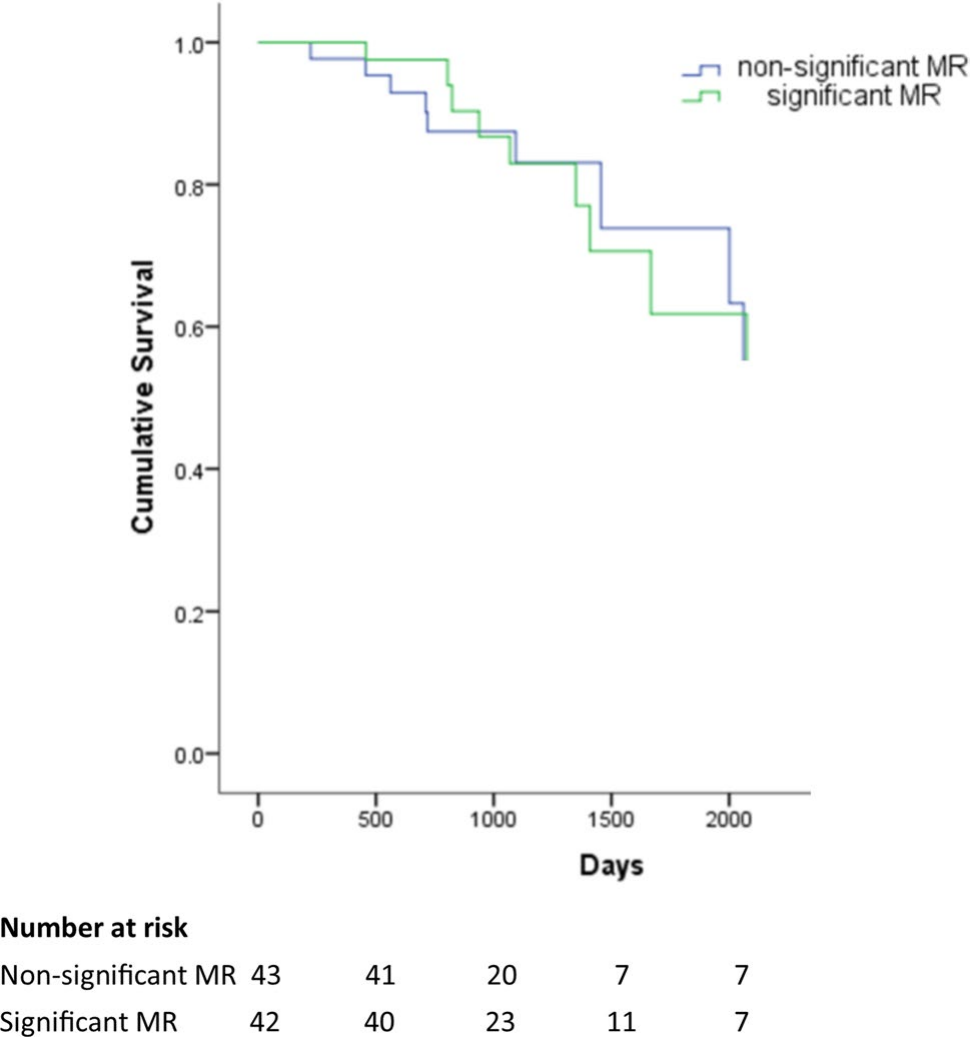
Kaplan Meier Curve for cumulative survival in ‘significant’ and ‘non-significant MR’ groups according to baseline status. Log rank p = 0.94. *MR* mitral regurgitation

At a median of 3 (IQR 2.03–3.97) years follow-up, 24% (n = 20) of TAVR patients had died. MR severity at baseline did not differ between those who died and those who did not; (mortality rate 13% vs 14%, non-significant vs significant, p = 0.84) (Fig. 4). Those who died also had a comparable reduction in MR severity post-TAVR (− 7.3% vs − 8.3%, p = 0.81). Cumulative survival rates between the ‘improvers’ and ‘non-improvers’ did not differ at follow up (mean survival 5.5 years 95% CI 4.6–6.4 vs 5.5 years 95% CI 4.7–6.3, improvers vs non-improvers). Residual significant MR was also not associated with increased mortality.

Intra-observer variability for LV quantitation in this study was 1.6%, 3.6%, 3.0% and 1.8% for LV end-diastolic volume, LV mass, LV stroke volume and LV ejection fraction respectively; whilst the coefficient of variation for peak aortic flow velocity and aortic forward flow volume was 0.2% and 1.7%.

## Discussion

This is the first CMR study to specifically assess MR in quantitative terms and evaluate its impact on cardiac reverse remodeling and mortality in patients undergoing TAVR. The main findings were (1) MR was shown to occur frequently in a TAVR population and those with ‘significant MR’ had a greater LV mass at baseline; (2) The presence of significant MR at baseline did not prevent LV reverse remodeling, as demonstrated by the substantial reduction in LV mass, LV diastolic and systolic volumes; (3) In those with significant MR at baseline, the MR is likely to improve following TAVR without the need for any specific intervention on the mitral valve; (4) The presence of LGE at baseline was associated with a greater improvement in MR at 6-months post-TAVR; (5) Improvement in MR was neither associated with lower mortality nor more favourable cardiac reverse remodeling compared with the ‘non-improvers’; (6) Baseline MR severity was not associated with long-term mortality.

Our findings are consistent with other large echocardiographic registries such as the Canadian Edwards SAPIEN registry [5], Italian CoreValve registry [14], and PARTNER Trial Cohort A study [15] demonstrating that TAVR is associated with a significant amelioration in MR severity. Although some studies suggested that significant MR results in an increase in mortality rates after TAVR [5, 14, 16, 17], the findings in our study are consistent with others [4, 7, 15, 18] which have not confirmed this association. Patients with a greater LV mass at baseline and higher aortic valve velocities (i.e. pressure-loaded ventricles) had a higher degree of MR in our study, likely due to raised LVEDP. We postulate that TAVR leads to the reduction of LVEDP and subsequently results in the amelioration of MR.

Interestingly, we found that improvement in MR was neither associated with more favourable cardiac reverse remodeling nor lower mortality rates compared with the ‘non-improvers’. There is however the possibility that a 6-month follow-up scan may have been too early to identify any difference in reverse remodeling between the groups. We also found that the presence of LGE at baseline was associated with improvement in MR 6-months post-TAVR. A possible explanation is that patients with significant MR tend to have a more critical AS and a higher trans-valvular gradient, which inevitably results in a higher LV mass and myocardial replacement fibrosis, depicted as LGE. The greater alleviation of ventricular afterload in these patients following TAVR could result in greater LV mass regression and systolic atrioventricular gradient, leading to a greater degree of MR reduction.

A key strength of our study was the use of CMR to reliably quantitate MR volume with low intra- and inter-observer variabilities, irrespective of MR jet geometry [9, 19]. Previous TAVR studies have frequently used transthoracic echocardiography for MR assessment, which has limited reproducibility and relies on mathematical assumptions of LV geometry and cavity size, which may not apply in the remodeled ventricle. In fact, echocardiography, when compared to CMR, was found to overestimate MR severity in many patients [20]. Some studies have also suggested that CMR is more accurate than echocardiography in assessing the severity of MR, especially in those with prolapsing leaflets and eccentric jets [21]. Echocardiographic evaluation of MR severity requires integration of various qualitative and quantitative measurements [22]. The variety of methods used for the quantitative assessment of MR may further explain the discrepancies amongst previous studies [5, 15–18].

The presence of myocardial fibrosis has been reported to be an adverse prognostic marker in patients with AS, with a 6–8 fold increased mortality risk [23, 24]. Myocardial fibrosis has also been shown to adversely affect prognosis and functional outcomes following surgical aortic valve replacement [13], but as yet its role is not fully elucidated in a TAVR population. In a small study (n = 20), the presence of LGE was found to predict higher cardiovascular mortality in patients with severe AS undergoing trans-femoral TAVR [25]. The clinical impact of LGE, however, has never been assessed in the setting of concomitant MR in severe AS. We have shown that the presence of LGE was associated with an improvement in MR in the short term (6 months) following TAVR, although the mechanism for this remains undefined.

Despite excellent procedural success and outcomes following TAVR, issues remain regarding optimal patient selection. Decision-making in patients with significant MR in the context of severe AS is often complex. One option is to perform a double valve (aortic and mitral) surgical procedure, which might be considered too high-risk in this already high-risk population. The other option is to perform TAVR as a compromise solution, accepting non-treatment of concomitant MR with a potential negative impact on patient outcomes. Therefore, identifying patients with the highest and lowest likelihood for MR improvement could be very important in the clinical decision-making process. LGE-CMR might allow clinicians to select patients who will most benefit from the TAVR procedure, obviating the need for high-risk double valve surgery. On the other hand, double-valve surgery may be more appropriate in patients with a low likelihood of MR improvement after TAVR. Although our small sample size did not demonstrate mortality benefits in those who improved their MR status, the literature to date has shown that MR improvement contributes to patient symptomatic improvement [26–28].

### Limitations

The moderate sample size, short follow-up time frame and the single-centre study design limits the strength of our conclusions. However, comparisons between the two groups using the highly reproducible technique of CMR meant it was appropriately powered for LV reverse remodeling parameters. The exclusion of patients with pacemakers (7%), severe AR and inclusion of survivors only in the CMR analysis raises the potential for selection bias. The analyzed population however did not differ in terms of baseline characteristics from the original whole study population. Because we excluded patients with contraindications to CMR and specific medical conditions, our study population is highly selected and so our conclusions cannot be extrapolated to all patients with severe AS.

Additionally, our study had a high proportion of patients with atrial fibrillation (20%), an arrhythmia which could reduce the quality of image acquisition and therefore reduce the accuracy of volumetric quantification with CMR. MR fraction in the context of severe AS may be overestimated using CMR phase contrast imaging due to underestimation of aortic forward flow when sampling high velocities. When performing phase contrast-based flow measurements in patients with heart valve replacement, there is also a potential for flow and volume miscalculation due to prosthesis-related distortions of the magnetic field [29]. Confounders such as primary or ischemic etiology, change in medications and development of bundle branch block or aortic regurgitation could additionally impact on cardiac reverse remodeling following TAVR. Finally, quantification of fibrosis on LGE images were analyzed using a semi-automatic, signal intensity threshold method rather than the newer T1 mapping techniques, as the latter were not widely employed at the time of patient recruitment.

## Conclusion

Significant MR is common in patients undergoing TAVR and improves in the majority post-procedure. Improvement in MR was not associated with LV reverse remodeling and baseline MR severity was not associated with mortality.

## Electronic supplementary material

Below is the link to the electronic supplementary material.

Supplementary material 1 (DOCX 19 KB)

## Abbreviations

AS: Aortic stenosis
AVA: Aortic valve area
CMR: Cardiovascular magnetic resonance
FOV: Field of view
LGE: Late gadolinium enhancement
LV: Left ventricular
LVEDP: LV end-diastolic pressure
MR: Mitral regurgitation
PG: Pressure gradient
RV: Right ventricular
SD: Standard deviation
TAVR: Transcatheter aortic valve replacement
VENC: Velocity encoded
