# Supplementary material for: CMR quantitation of change in mitral regurgitation following transcatheter aortic valve replacement (TAVR): impact on left ventricular reverse remodeling and outcome
Source: Int J Cardiovasc Imaging. 2018 Sep 4;35(1):161–70. doi: 10.1007/s10554-018-1441-y (PMC6373302; doi:10.1007/s10554-018-1441-y)
Supplement: Supplementary file 1 — Supplementary material 1 (DOCX 19 KB) [file 10554_2018_1441_MOESM1_ESM.docx]

CMR quantitation of change in Mitral Regurgitation following Transcatheter Aortic Valve Replacement (TAVR): Impact on left ventricular reverse remodeling and outcome

Pei G Chew, MBChB^1^, Laura E Dobson, MD^1^, Pankaj Garg, MD^1^, Timothy A Fairbairn, PhD^1^, Tarique A Musa, PhD^1^, Akhlaque Uddin, MD^1^, Peter P Swoboda, PhD^1^, James R Foley, MBChB^1^, Graham J Fent, MBChB^1^, Louise AE Brown, MBChB^1^, Sebastian Onciul, MBChB^1^, Sven Plein, PhD^1^, Daniel J Blackman^2^, John P Greenwood, PhD^1^.

^1^ Multidisciplinary Cardiovascular Research Centre (MCRC) & Leeds Institute of Cardiovascular and Metabolic Medicine (LICAMM), University of Leeds, United Kingdom ^2^ Leeds Teaching Hospitals NHS Trust, Leeds, UK

**Address for correspondence:**

Professor J.P. Greenwood

Multidisciplinary Cardiovascular Research Centre,

Leeds Institute for Cardiovascular and Metabolic Medicine,

University of Leeds, LS2 9JT, United Kingdom

Tel +44 113 39 22650

Fax +44 113 3922311

Email: j.greenwood@leeds.ac.uk

**SUPPLEMENTARY APPENDIX**

Table S1. Results of logistic regression for the improvement in MR post-TAVR

| **UNIVARIATE REGRESSION ANALYSIS**  **Visit 1** | **R** | **R^2^** | **F value** | **Standardised Co-efficient Beta** | **Beta CI**  **Lower** | **Beta CI**  **upper** | **Univariate**  **P value** |
| --- | --- | --- | --- | --- | --- | --- | --- |
| Sex | 0.08 | 0.006 | 0.55 | -0.08 | -9.89 | 4.5 | 0.46 |
| Age | 0.03 | 0.001 | 0.11 | 0.03 | -0.40 | 0.57 | 0.73 |
| Logistic score | 0.04 | 0.002 | 0.13 | -0.04 | -0.32 | 0.22 | 0.71 |
| Euro II score | 0.04 | 0.001 | 0.15 | 0.04 | -0.65 | 0.97 | 0.69 |
| STS mortality | 0.17 | 0.02 | 2.53 | -0.17 | -2.15 | 0.23 | 0.11 |
| STS morbidity | 0.09 | 0.01 | 0.78 | -0.09 | -0.61 | 0.23 | 0.37 |
| AF | 0.29 | 0.08 | 8.17 | -0.29 | -20.4 | -3.67 | 0.005 |
| MI | 0.12 | 0.014 | 1.23 | 0.12 | -3.78 | 13.3 | 0.27 |
| CABG | 0.18 | 0.04 | 3.05 | 0.18 | -0.93 | 14.5 | 0.08 |
| PHT | 0.003 | <0.001 | 0.001 | -0.003 | -7.5 | 7.3 | 0.98 |
| Change in LVEDP | 0.20 | 0.04 | 3.17 | 0.21 | -0.07 | 1.29 | 0.07 |
| **CMR characteristics** |  |  |  |  |  |  |  |
| LV Mass (g) | 0.14 | 0.02 | 1.73 | 0.14 | -0.03 | 0.16 | 0.19 |
| LVEDV (ml) | 0.05 | 0.003 | 0.27 | 0.05 | -0.05 | 0.09 | 0.59 |
| LVESV (ml) | 0.006 | < 0.001 | 0.003 | 0.006 | -0.08 | 0.08 | 0.95 |
| LVSV (ml) | 0.11 | 0.013 | 1.09 | 0.114 | -0.07 | 0.24 | 0.29 |
| LVEF (%) | 0.02 | <0.001 | 0.05 | 0.02 | -0.25 | 0.33 | 0.80 |
| RVEDV (ml) | 0.07 | 0.005 | 0.46 | 0.07 | -0.06 | 0.13 | 0.49 |
| RVESV (ml) | 0.038 | 0.001 | 0.123 | -0.038 | -0.16 | 0.11 | 0.72 |
| RVSV (ml) | 0.20 | 0.04 | 3.59 | 0.20 | -0.008 | 0.37 | 0.06 |
| RVSVi (ml/m^2^) | 0.15 | 0.02 | 2.15 | 0.15 | -0.09 | 0.61 | 0.14 |
| RVEF(%) | 0.18 | 0.03 | 2.89 | 0.18 | -0.05 | 0.68 | 0.09 |
| LA Volumes (ml) | 0.06 | 0.004 | 0.33 | -0.06 | -0.10 | 0.05 | 0.56 |
| **Aortic valve parameters (CMR)** |  |  |  |  |  |  |  |
| Aortic FF (ml) | -0.338 | 0.013 | 11.4 | -0.34 | -0.49 | -0.13 | 0.001 |
| Ao Volume | 0.45 | 0.20 | 12.17 | -0.45 | -0.8 | -0.21 | 0.001 |
| Ao Rfraction (ml) | 0.35 | 0.12 | 11.8 | 0.35 | 0.32 | 1.22 | <0.001 |
| Aortic max PG (mmHg) | 0.17 | 0.028 | 2.44 | 0.17 | -0.04 | 0.39 | 0.12 |
| Mean gradient, (mmHg) | 0.21 | 0.04 | 2.12 | 0.21 | -0.32 | 2.03 | 0.15 |
| Ao peak vel (m/s) | 0.20 | 0.04 | 2.08 | 0.20 | -0.01 | 0.10 | 0.15 |
| MR RVol (ml) | 0.56 | 0.32 | 39.1 | 0.56 | 0.42 | 0.82 | <0.001 |
| MR Rfraction (%) | 0.66 | 0.44 | 67.3 | 0.66 | 0.63 | 1.03 | <0.001 |
| **VISIT 2**  **(Follow-up)** |  |  |  |  |  |  |  |
| **Aortic valve parameters (CMR)** |  |  |  |  |  |  |  |
| Ao FF (ml) | 0.39 | 0.15 | 15.1 | 0.39 | 0.18 | 0.56 | <0.001 |
| Ao Volume | 0.46 | 0.21 | 13.62 | 0.46 | 0.19 | 0.65 | <0.001 |
| Ao Rfraction (ml) | 0.16 | 0.02 | 2.31 | -0.16 | -1.04 | 0.13 | 0.132 |
| Ao max PG (mmHg) | 0.29 | 0.08 | 7.88 | 0.29 | 0.133 | 0.77 | 0.006 |
| Mean gradient, (mmHg) | 0.26 | 0.07 | 3.79 | 0.26 | -0.04 | 2.77 | 0.05 |
| Ao peak velocity (m/s) | 0.32 | 0.10 | 5.6 | 0.32 | 0.01 | 0.16 | 0.02 |
| RVSV (ml) | 0.18 | 0.03 | 3.05 | 0.18 | -0.02 | 0.32 | 0.08 |
| RVEF(%) | 0.27 | 0.07 | 6.58 | 0.27 | 0.09 | 0.78 | 0.01 |

AF, atrial fibrillation; CABG, coronary artery bypass graft; FF, forward flow; LA, left atrial; LVEDP, left ventricular end diastolic pressure; LVEDV, left ventricular end-diastolic volume; LVEF, left ventricular ejection fraction; LVESV, left ventricular end-systolic volume; LVSV, left ventricular stroke volume; MI, myocardial infarction; MR, mitral regurgitant; PG, pressure gradient; PHT, pulmonary hypertension; RFraction, regurgitant fraction; RVEDV, right ventricular end-diastolic volume; RVEF, right ventricular ejection fraction; RVESV, right ventricular end-systolic volume; RVol, regurgitant volume; RVSV, right ventricular stroke volume; RVSVi, right ventricular stroke volume(indexed); STS, Society of Thoracic Surgery
